# Supplementary material for: Congenital Zika Syndrome: Insights from Integrated Proteomic and Metabolomic Analysis
Source: Biomolecules. 2024 Dec 30;15(1):32. doi: 10.3390/biom15010032 (PMC11762526; doi:10.3390/biom15010032)
Supplement: Supplementary file 1 [file biomolecules-15-00032-s001.zip › biomolecules-3172539-supplementary.pdf]

Supplementary Material Table S1 - Proteomics Analysis: Access, Peptides, Scores and Normalized Abundances

Accession	Peptides	Score	Anova (p)*	Fold	Description	Average Normalised Abundances	
						CZS-	CZS+
**P02768**	349 (293)	1729.83	5.00e-003	1.39	Albumin OS=Homo sapiens OX=9606 GN=ALB PE=1 SV=2	1225.17	882.65
**P00489**	147 (61)	1460.11	0.01	1.22	Glycogen phosphorylase_ muscle form OS=Oryctolagus cuniculus OX=9986 GN=PYGM PE=1 SV=3	193.31	236.65

Supplementary Material Table S1 - Proteomics Analysis: Access, Peptides, Scores and Normalized Abundances

**P01023**	163 (121)	1322.14	0.78	1.07	Alpha-2-macroglobulin OS=Homo sapiens OX=9606 GN=A2M PE=1 SV=3	50.90	47.43
**P01024**	133 (104)	1171.58	0.05	1.40	Complement C3 OS=Homo sapiens OX=9606 GN=C3 PE=1 SV=2	141.24	197.45

**P11217**	151 (59)	1168.39	0.24	1.42	Glycogen phosphorylase_ muscle form OS=Homo sapiens OX=9606 GN=PYGM PE=1 SV=6	34.33	48.61
**P02787**	104 (87)	897.65	0.01	1.42	Serotransferrin OS=Homo sapiens OX=9606 GN=TF PE=1 SV=3	52.62	37.10

**P02675**	73 (61)	657.65	0.12	1.14	Fibrinogen beta chain OS=Homo sapiens OX=9606 GN=FGB PE=1 SV=2	112.59	98.86
**P02671**	75 (52)	594.66	0.15	1.21	Fibrinogen alpha chain OS=Homo sapiens OX=9606 GN=FGA PE=1 SV=2	40.51	33.48
**P02647**	59 (51)	550.40	7.61e-004	1.64	Apolipoprotein A-I OS=Homo sapiens OX=9606 GN=APOA1 PE=1 SV=1	102.44	62.44

Supplementary Material Table S1 - Proteomics Analysis: Access, Peptides, Scores and Normalized Abundances

**P11216**	48 (22)	542.31	0.86	1.07	Glycogen phosphorylase_ brain form OS=Homo sapiens OX=9606 GN=PYGB PE=1 SV=5	24.63	26.35
**P00738**	68 (39)	541.81	0.30	1.01	Haptoglobin OS=Homo sapiens OX=9606 GN=HP PE=1 SV=1	105.70	107.14

Supplementary Material Table S1 - Proteomics Analysis: Access, Peptides, Scores and Normalized Abundances

**P01871**	78 (59)	533.30	0.18	1.16	Immunoglobulin heavy constant mu OS=Homo sapiens OX=9606 GN=IGHM PE=1 SV=4	139.57	120.72
**P01009**	71 (53)	532.33	0.36	1.03	Alpha-1-antitrypsin OS=Homo sapiens OX=9606 GN=SERPINA1 PE=1 SV=3	90.15	92.49

Supplementary Material Table *S*1 - Proteomics Analysis: Access, Peptides, Scores and Normalized Abundances

**P00450**	50 (42)	462.77	0.90	1.39	Ceruloplasmin OS=Homo sapiens OX=9606 GN=CP PE=1 SV=1	14.82	20.64
**P02679**	46 (30)	437.13	0.12	1.26	Fibrinogen gamma chain OS=Homo sapiens OX=9606 GN=FGG PE=1 SV=3	79.67	63.43
**P06737**	38 (20)	372.27	0.06	1.51		35.55	53.85

Supplementary Material Table *S*1 - Proteomics Analysis: Access, Peptides, Scores and Normalized Abundances

					Glycogen phosphorylase_ liver form OS=Homo sapiens OX=9606 GN=PYGL PE=1 SV=4		
**P0C0L4**	41 (2)	367.20	1.74e-003	1.26	Complement C4-A OS=Homo sapiens OX=9606 GN=C4A PE=1 SV=2	58.66	73.70
**P0C0L5**	40 (1)	353.61	4.49e-003	1.26	Complement C4-B OS=Homo sapiens OX=9606 GN=C4B PE=1 SV=2	0.45	0.57

Supplementary Material Table ***S*1** - Proteomics Analysis: Access, Peptides, Scores and Normalized Abundances

**P04264**	37 (27)	343.18	9.61e-003	1.22	Keratin_ type II cytoskeletal 1 OS=Homo sapiens OX=9606 GN=KRT1 PE=1 SV=6		11.92	14.57
**P02790**	34 (27)	332.40	2.32e-003	1.31	Hemopexin OS=Homo sapiens OX=9606 GN=HPX PE=1 SV=2		41.14	31.40

Supplementary Material Table S1 - Proteomics Analysis: Access, Peptides, Scores and Normalized Abundances

**P01860**	33 (14)	325.55	4.67e-005	1.58	Immunoglobulin heavy constant gamma 3 OS=Homo sapiens OX=9606 GN=IGHG3 PE=1 SV=2		13.83	8.74
**P20742**	45 (26)	321.71	0.99	1.01	Pregnancy zone protein OS=Homo sapiens OX=9606 GN=PZP PE=1 SV=4		12.85	12.72

Supplementary Material Table S1 - Proteomics Analysis: Access, Peptides, Scores and Normalized Abundances

**P00739**	26 (7)	311.06	0.13	1.14	Haptoglobin-related protein OS=Homo sapiens OX=9606 GN=HPR PE=2 SV=2		17.93	15.76
**P01857**	39 (21)	306.71	1.01e-003	1.50	Immunoglobulin heavy constant gamma 1 OS=Homo sapiens OX=9606 GN=IGHG1 PE=1 SV=1		237.66	158.28

Supplementary Material Table S1 - Proteomics Analysis: Access, Peptides, Scores and Normalized Abundances

**P02774**	33 (28)	267.80	0.29	1.09	Vitamin D-binding protein OS=Homo sapiens OX=9606 GN=GC PE=1 SV=2		5.70	6.19
**P13645**	27 (19)	264.18	0.66	1.08	Keratin_ type I cytoskeletal 10 OS=Homo sapiens OX=9606 GN=KRT10 PE=1 SV=6		8.64	9.31

Supplementary Material Table S1 - Proteomics Analysis: Access, Peptides, Scores and Normalized Abundances

**P01859**	46 (23)	257.51	1.84e-005	1.81	Immunoglobulin heavy constant gamma 2 OS=Homo sapiens OX=9606 GN=IGHG2 PE=1 SV=2	63.12	34.92
**P01876**	22 (12)	252.54	3.59e-004	1.40	Immunoglobulin heavy constant alpha 1 OS=Homo sapiens OX=9606 GN=IGHA1 PE=1 SV=2	42.81	30.63

Supplementary Material Table $S1$ - Proteomics Analysis: Access, Peptides, Scores and Normalized Abundances

**P35908**	26 (14)	232.14	0.92	1.18	Keratin_ type II cytoskeletal 2 epidermal OS=Homo sapiens OX=9606 GN=KRT2 PE=1 SV=2	6.76	7.99
**P01011**	25 (19)	218.45	7.01e-004	1.45	Alpha-1-antichymotrypsin OS=Homo sapiens OX=9606 GN=SERPINA3 PE=1 SV=2	74.03	51.17

Supplementary Material Table S1 - Proteomics Analysis: Access, Peptides, Scores and Normalized Abundances

**P01861**	19 (9)	215.77	2.21e-006	1.77	Immunoglobulin heavy constant gamma 4 OS=Homo sapiens OX=9606 GN=IGHG4 PE=1 SV=1	22.56	12.76
**P05155**	23 (18)	214.95	0.01	1.29	Plasma protease C1 inhibitor OS=Homo sapiens OX=9606 GN=SERPING1 PE=1 SV=2	16.04	12.44

Supplementary Material Table S1 - Proteomics Analysis: Access, Peptides, Scores and Normalized Abundances

**P02751**	30 (28)	213.19	0.47	1.11	Fibronectin OS=Homo sapiens OX=9606 GN=FN1 PE=1 SV=5	9.99	9.03
**P68871**	11 (6)	171.41	0.44	1.04	Hemoglobin subunit beta OS=Homo sapiens OX=9606 GN=HBB PE=1 SV=2	23.28	24.19

Supplementary Material Table S1 - Proteomics Analysis: Access, Peptides, Scores and Normalized Abundances

**P01834**	22 (20)	171.19	2.05e-004	1.50	Immunoglobulin kappa constant OS=Homo sapiens OX=9606 GN=IGKC PE=1 SV=2	246.68	164.36
**P02766**	13 (10)	171.05	2.65e-004	1.71	Transthyretin OS=Homo sapiens OX=9606 GN=TTR PE=1 SV=1	10.60	6.20
**P01008**	21 (17)	168.61	0.02	1.18		7.11	6.01

Supplementary Material Table $S1$ - Proteomics Analysis: Access, Peptides, Scores and Normalized Abundances

					Antithrombin-III OS=Homo sapiens OX=9606 GN=SERPINC1 PE=1 SV=1		
**P02763**	14 (7)	160.55	1.35e-006	2.17	Alpha-1-acid glycoprotein 1 OS=Homo sapiens OX=9606 GN=ORM1 PE=1 SV=1	22.88	10.56
**P60709**	16 (1)	155.44	0.90	1.01		0.27	0.27

Supplementary Material Table $S1$ - Proteomics Analysis: Access, Peptides, Scores and Normalized Abundances

					Actin_ cytoplasmic 1 OS=Homo sapiens OX=9606 GN=ACTB PE=1 SV=1		
**B9A064**	13 (6)	155.40	2.88e-003	1.33	Immunoglobulin lambda-like polypeptide 5 OS=Homo sapiens OX=9606 GN=IGLL5 PE=2 SV=2	32.45	24.38

Supplementary Material Table S1 - Proteomics Analysis: Access, Peptides, Scores and Normalized Abundances

**P04003**	20 (12)	152.97	0.04	1.18	C4b-binding protein alpha chain OS=Homo sapiens OX=9606 GN=C4BPA PE=1 SV=2	35.98	42.33
**P63261**	16 (1)	150.09	0.05	1.19	Actin_ cytoplasmic 2 OS=Homo sapiens OX=9606 GN=ACTG1 PE=1 SV=1	8.62	7.23

Supplementary Material Table $S1$ - Proteomics Analysis: Access, Peptides, Scores and Normalized Abundances

**P0DOY3**	9 (3)	138.84	0.42	1.01	Immunoglobulin lambda constant 3 OS=Homo sapiens OX=9606 GN=IGLC3 PE=1 SV=1	2.86	2.83
**P01877**	14 (4)	132.47	0.61	1.00	Immunoglobulin heavy constant alpha 2 OS=Homo sapiens OX=9606 GN=IGHA2 PE=1 SV=4	32.11	32.07

**P0DOY2**	9 (0)	132.12	---	---	Immunoglobulin lambda constant 2 OS=Homo sapiens OX=9606 GN=IGLC2 PE=1 SV=1	---	---
**P10909**	16 (8)	130.97	0.05	1.15	Clusterin OS=Homo sapiens OX=9606 GN=CLU PE=1 SV=1	13.04	11.34

Supplementary Material Table S1 - Proteomics Analysis: Access, Peptides, Scores and Normalized Abundances

**P19652**	12 (7)	129.48	2.18e-006	1.68	Alpha-1-acid glycoprotein 2 OS=Homo sapiens OX=9606 GN=ORM2 PE=1 SV=2	5.60	3.34
**P02649**	14 (10)	121.30	1.58e-003	1.37	Apolipoprotein E OS=Homo sapiens OX=9606 GN=APOE PE=1 SV=1	10.82	7.92
**P02749**	12 (9)	113.33	1.57e-004	2.66		9.66	3.63

Supplementary Material Table S1 - Proteomics Analysis: Access, Peptides, Scores and Normalized Abundances

					Beta-2-glycoprotein 1 OS=Homo sapiens OX=9606 GN=APOH PE=1 SV=3		
**P0CF74**	15 (8)	106.12	4.64e-003	1.31	Immunoglobulin lambda constant 6 OS=Homo sapiens OX=9606 GN=IGLC6 PE=1 SV=1	5.15	3.94

Supplementary Material Table S1 - Proteomics Analysis: Access, Peptides, Scores and Normalized Abundances

**P02765**	12 (8)	105.55	0.84	1.05	Alpha-2-HS-glycoprotein OS=Homo sapiens OX=9606 GN=AHSG PE=1 SV=2	11.93	11.34
**P62736**	10 (1)	101.74	0.45	1.09	Actin_ aortic smooth muscle OS=Homo sapiens OX=9606 GN=ACTA2 PE=1 SV=1	0.35	0.38

**P35527**	12 (7)	96.98	3.22e-003	1.88	Keratin_ type I cytoskeletal 9 OS=Homo sapiens OX=9606 GN=KRT9 PE=1 SV=3	1.15	2.18
**P01772**	8 (1)	96.26	0.23	84.62	Immunoglobulin heavy variable 3-33 OS=Homo sapiens OX=9606 GN=IGHV3-33 PE=1 SV=2	7.83e-005	6.63e-003

Supplementary Material Table *S*1 - Proteomics Analysis: Access, Peptides, Scores and Normalized Abundances

**P02652**	11 (9)	95.03	0.28	1.15	Apolipoprotein A-II OS=Homo sapiens OX=9606 GN=APOA2 PE=1 SV=1	7.36	6.38
**P02042**	9 (2)	91.71	0.87	1.01	Hemoglobin subunit delta OS=Homo sapiens OX=9606 GN=HBD PE=1 SV=2	2.14	2.16
**P01019**	7 (6)	86.63	0.38	1.07	Angiotensinogen OS=Homo sapiens OX=9606 GN=AGT PE=1 SV=1	3.97	3.71

Supplementary Material Table $S1$ - Proteomics Analysis: Access, Peptides, Scores and Normalized Abundances

**P01780**	6 (1)	85.70	0.01	4.63	Immunoglobulin heavy variable 3-7 OS=Homo sapiens OX=9606 GN=IGHV3-7 PE=1 SV=2	0.16	0.03
**P01767**	7 (1)	84.82	1.57e-003	4.40	Immunoglobulin heavy variable 3-53 OS=Homo sapiens OX=9606 GN=IGHV3-53 PE=1 SV=2	0.07	0.02

Supplementary Material Table S1 - Proteomics Analysis: Access, Peptides, Scores and Normalized Abundances

**P08603**	12 (8)	84.60	0.37	1.21	Complement factor H OS=Homo sapiens OX=9606 GN=CFH PE=1 SV=4	1.48	1.79
**P01591**	11 (8)	84.38	0.29	1.04	Immunoglobulin J chain OS=Homo sapiens OX=9606 GN=JCHAIN PE=1 SV=4	3.94	4.10

Supplementary Material Table ***S*****1** - Proteomics Analysis: Access, Peptides, Scores and Normalized Abundances

**Q6S8J3**	12 (6)	84.29	5.00e-003	1.24	POTE ankyrin domain family member E OS=Homo sapiens OX=9606 GN=POTEE PE=2 SV=3	5.74	4.62
**P01768**	6 (0)	81.44	---	---	Immunoglobulin heavy variable 3-30 OS=Homo sapiens OX=9606 GN=IGHV3-30 PE=1 SV=2	---	---

Supplementary Material Table S1 - Proteomics Analysis: Access, Peptides, Scores and Normalized Abundances

**A0M8Q6**	6 (2)	78.74	4.86e-004	1.67	Immunoglobulin lambda constant 7 OS=Homo sapiens OX=9606 GN=IGLC7 PE=1 SV=3	1.09	0.65
**P0DP02**	6 (1)	77.28	0.28	1.23	Immunoglobulin heavy variable 3-30-3 OS=Homo sapiens OX=9606 GN=IGHV3-30-3 PE=3 SV=1	0.96	1.18

Supplementary Material Table ***S*1** - Proteomics Analysis: Access, Peptides, Scores and Normalized Abundances

**P69905**	11 (7)	75.48	0.34	1.02	Hemoglobin subunit alpha OS=Homo sapiens OX=9606 GN=HBA1 PE=1 SV=2	10.60	10.41
**A0A0B4J1X5**	5 (1)	72.02	0.01	1.58	Immunoglobulin heavy variable 3-74 OS=Homo sapiens OX=9606 GN=IGHV3-74 PE=3 SV=1	0.58	0.37

Supplementary Material Table S1 - Proteomics Analysis: Access, Peptides, Scores and Normalized Abundances

**O43866**	10 (8)	70.11	0.15	1.06	CD5 antigen-like OS=Homo sapiens OX=9606 GN=CD5L PE=1 SV=1	3.80	3.59
**P01764**	5 (1)	69.13	0.05	1.66	Immunoglobulin heavy variable 3-23 OS=Homo sapiens OX=9606 GN=IGHV3-23 PE=1 SV=2	0.34	0.21

**P00751**	9 (7)	68.72	3.85e-005	1.98	Complement factor B OS=Homo sapiens OX=9606 GN=CFB PE=1 SV=2	2.16	1.09
**Q08380**	8 (5)	62.32	0.02	1.19	Galectin-3-binding protein OS=Homo sapiens OX=9606 GN=LGALS3BP PE=1 SV=1	2.15	1.80

Supplementary Material Table S1 - Proteomics Analysis: Access, Peptides, Scores and Normalized Abundances

**A0A0B4J1V1**	5 (0)	61.90	---	---	Immunoglobulin heavy variable 3-21 OS=Homo sapiens OX=9606 GN=IGHV3-21 PE=1 SV=1	---	---
**P01042**	9 (8)	61.16	0.54	1.04	Kininogen-1 OS=Homo sapiens OX=9606 GN=KNG1 PE=1 SV=2	4.00	4.17
**P04004**	8 (8)	60.32	0.07	1.13	Vitronectin OS=Homo sapiens OX=9606 GN=VTN PE=1 SV=1	4.63	4.09

Supplementary Material Table S1 - Proteomics Analysis: Access, Peptides, Scores and Normalized Abundances

**P04217**	9 (5)	59.73	0.02	1.26	Alpha-1B-glycoprotein OS=Homo sapiens OX=9606 GN=A1BG PE=1 SV=4		3.11	2.48
**P04259**	6 (2)	56.73	0.02	1.31	Keratin_ type II cytoskeletal 6B OS=Homo sapiens OX=9606 GN=KRT6B PE=1 SV=5		7.73	5.92

Supplementary Material Table S1 - Proteomics Analysis: Access, Peptides, Scores and Normalized Abundances

**P0DP06**	4 (4)	51.47	0.04	1.13	Immunoglobulin heavy variable 4-30-4 OS=Homo sapiens OX=9606 GN=IGHV4-30-4 PE=3 SV=1	7.90	6.99
**Q7Z794**	5 (1)	50.70	0.01	1.33	Keratin_ type II cytoskeletal 1b OS=Homo sapiens OX=9606 GN=KRT77 PE=1 SV=3	4.35	5.79

Supplementary Material Table S1 - Proteomics Analysis: Access, Peptides, Scores and Normalized Abundances

**O14791**	4 (2)	49.20	0.41	1.45	Apolipoprotein L1 OS=Homo sapiens OX=9606 GN=APOL1 PE=1 SV=5	0.18	0.26
**P06727**	7 (6)	45.07	0.10	1.10	Apolipoprotein A-IV OS=Homo sapiens OX=9606 GN=APOA4 PE=1 SV=4	2.97	2.71
**P06312**	3 (2)	39.18	3.97e-003	1.36	Immunoglobulin kappa variable 4-1 OS=Homo sapiens OX=9606 GN=IGKV4-1 PE=1 SV=1	4.52	3.33

Supplementary Material Table S1 - Proteomics Analysis: Access, Peptides, Scores and Normalized Abundances

**P27169**	6 (5)	38.33	0.64	1.22	Serum paraoxonase/arylesterase 1 OS=Homo sapiens OX=9606 GN=PON1 PE=1 SV=3	5.83	7.12
**P01782**	3 (1)	36.34	1.31e-003	21.09	Immunoglobulin heavy variable 3-9 OS=Homo sapiens OX=9606 GN=IGHV3-9 PE=1 SV=2	0.04	1.88e-003

Supplementary Material Table $S1$ - Proteomics Analysis: Access, Peptides, Scores and Normalized Abundances

**P01619**	4 (3)	35.94	4.74e-003	1.26	Immunoglobulin kappa variable 3-20 OS=Homo sapiens OX=9606 GN=IGKV3-20 PE=1 SV=2	6.01	4.76
**P25311**	4 (2)	33.50	4.63e-006	2.21	Zinc-alpha-2-glycoprotein OS=Homo sapiens OX=9606 GN=AZGP1 PE=1 SV=2	1.86	0.84

Supplementary Material Table S1 - Proteomics Analysis: Access, Peptides, Scores and Normalized Abundances

**P08697**	7 (5)	33.20	5.79e-003	1.31	Alpha-2-antiplasmin OS=Homo sapiens OX=9606 GN=SERPINF2 PE=1 SV=3	3.15	2.40
**A0A0J9YVY3**	2 (1)	31.94	0.68	1.02	Immunoglobulin heavy variable 7-4-1 OS=Homo sapiens OX=9606 GN=IGHV7-4-1 PE=3 SV=1	0.96	0.94

Supplementary Material Table $S1$ - Proteomics Analysis: Access, Peptides, Scores and Normalized Abundances

**P04196**	4 (4)	31.24	0.90	1.01	Histidine-rich glycoprotein OS=Homo sapiens OX=9606 GN=HRG PE=1 SV=1	2.82	2.79
**P01602**	3 (2)	29.78	0.01	1.24	Immunoglobulin kappa variable 1-5 OS=Homo sapiens OX=9606 GN=IGKV1-5 PE=1 SV=2	3.03	2.44

Supplementary Material Table S1 - Proteomics Analysis: Access, Peptides, Scores and Normalized Abundances

**Q03014**	4 (3)	29.77	1.53e-004	1.59	Hematopoietically-expressed homeobox protein HHEX OS=Homo sapiens OX=9606 GN=HHEX PE=1 SV=1	3.55	2.23
**Q14624**	5 (4)	29.77	3.53e-004	1.48	Inter-alpha-trypsin inhibitor heavy chain H4 OS=Homo sapiens OX=9606 GN=ITIH4 PE=1 SV=4	4.12	2.80

Supplementary Material Table S1 - Proteomics Analysis: Access, Peptides, Scores and Normalized Abundances

**P01700**	3 (3)	29.02	0.12	1.11	Immunoglobulin lambda variable 1-47 OS=Homo sapiens OX=9606 GN=IGLV1-47 PE=1 SV=2	5.42	4.90
**P02533**	3 (0)	28.07	---	---	Keratin_ type I cytoskeletal 14 OS=Homo sapiens OX=9606 GN=KRT14 PE=1 SV=4	---	---

Supplementary Material Table ***S*1** - Proteomics Analysis: Access, Peptides, Scores and Normalized Abundances

**A0A0B4J1V6**	3 (2)	27.25	0.48	1.00	Immunoglobulin heavy variable 3-73 OS=Homo sapiens OX=9606 GN=IGHV3-73 PE=3 SV=1			21.33	21.25
**Q9BYX7**	2 (1)	25.44	0.02	1.52	Putative beta-actin-like protein 3 OS=Homo sapiens OX=9606 GN=POTEKP PE=5 SV=1			28.61	18.86

Supplementary Material Table S1 - Proteomics Analysis: Access, Peptides, Scores and Normalized Abundances

**Q9BYC8**	2 (1)	25.34	6.47e-006	3.64	39S ribosomal protein L32_ mitochondrial OS=Homo sapiens OX=9606 GN=MRPL32 PE=1 SV=1	3.27	0.90
**Q6PKX4**	4 (1)	20.97	1.36e-003	1.42	Docking protein 6 OS=Homo sapiens OX=9606 GN=DOK6 PE=1 SV=1	11.57	8.12

**Q8WXB4**	3 (2)	19.79	0.54	1.53	Zinc finger protein 606 OS=Homo sapiens OX=9606 GN=ZNF606 PE=2 SV=1	3.60	5.51
**Q8TDH9**	2 (0)	19.77	---	---	Biogenesis of lysosome-related organelles complex 1 subunit 5 OS=Homo sapiens OX=9606 GN=BLOC1S5 PE=1 SV=1	---	---

Supplementary Material Table ***S*****1** - Proteomics Analysis: Access, Peptides, Scores and Normalized Abundances

**A0A0C4DH38**	2 (2)	19.43	1.84e-003	1.64	Immunoglobulin heavy variable 5-51 OS=Homo sapiens OX=9606 GN=IGHV5-51 PE=3 SV=1	2.10	1.28
**Q86X29**	3 (1)	19.35	0.08	1.24	Lipolysis-stimulated lipoprotein receptor OS=Homo sapiens OX=9606 GN=LSR PE=1 SV=4	178.44	143.52

Supplementary Material Table S1 - Proteomics Analysis: Access, Peptides, Scores and Normalized Abundances

**P02746**	2 (2)	18.81	0.06	1.16	Complement C1q subcomponent subunit B OS=Homo sapiens OX=9606 GN=C1QB PE=1 SV=3	1.01	0.87
**Q96D03**	3 (2)	18.44	1.09e-004	2.18	DNA damage-inducible transcript 4-like protein OS=Homo sapiens OX=9606 GN=DDIT4L PE=1 SV=1	4.25	1.96

Supplementary Material Table $S1$ **-** Proteomics Analysis: Access, Peptides, Scores and Normalized Abundances

**P00734**	3 (3)	18.38	3.09e-005	2.70	Prothrombin OS=Homo sapiens OX=9606 GN=F2 PE=1 SV=2	0.89	0.33
**Q8IXY8**	3 (3)	18.19	9.55e-005	1.48	Probable inactive peptidyl-prolyl cis-trans isomerase-like 6 OS=Homo sapiens OX=9606 GN=PPIL6 PE=1 SV=1	249.16	369.70

Supplementary Material Table *S*1 - Proteomics Analysis: Access, Peptides, Scores and Normalized Abundances

**P02656**	2 (1)	15.53	3.09e-005	19.21	Apolipoprotein C-III OS=Homo sapiens OX=9606 GN=APOC3 PE=1 SV=1	0.12	6.25e-003
**A0A0A0MRZ8**	2 (2)	14.80	1.08e-005	4.10	Immunoglobulin kappa variable 3D-11 OS=Homo sapiens OX=9606 GN=IGKV3D-11 PE=3 SV=6	0.45	0.11
**P05090**	2 (2)	14.19	2.00e-004	2.21	Apolipoprotein D OS=Homo sapiens OX=9606 GN=APOD PE=1 SV=1	0.18	0.08

**Q9HCL3**	1 (1)	13.86	8.40e-003	1.48	Zinc finger protein 14 homolog OS=Homo sapiens OX=9606 GN=ZFP14 PE=1 SV=2	11.41	7.72
**Q96M94**	1 (1)	13.69	2.92e-004	1.67	Kelch-like protein 15 OS=Homo sapiens OX=9606 GN=KLHL15 PE=1 SV=2	24.02	14.43

Supplementary Material Table S**1** - Proteomics Analysis: Access, Peptides, Scores and Normalized Abundances

**P02760**	2 (2)	13.65	2.09e-003	1.66	Protein AMBP OS=Homo sapiens OX=9606 GN=AMBP PE=1 SV=1	1.00	0.60
**Q6ZWK4**	1 (0)	13.37	---	---	Regulator of hemoglobinization and erythroid cell expansion protein OS=Homo sapiens OX=9606 GN=RHEX PE=1 SV=1	---	---

**Q8N1N4**	1 (0)	13.34	---	---	Keratin_ type II cytoskeletal 78 OS=Homo sapiens OX=9606 GN=KRT78 PE=1 SV=2	---	---
**Q9NZP8**	2 (0)	13.18	---	---	Complement C1r subcomponent-like protein OS=Homo sapiens OX=9606 GN=C1RL PE=1 SV=2	---	---

Supplementary Material Table S1 - Proteomics Analysis: Access, Peptides, Scores and Normalized Abundances

**Q96PD5**	2 (2)	13.16	0.79	1.11	N-acetylmuramoyl-L-alanine amidase OS=Homo sapiens OX=9606 GN=PGLYRP2 PE=1 SV=1	0.38	0.42
**P08887**	2 (1)	13.14	1.89e-007	3.29	Interleukin-6 receptor subunit alpha OS=Homo sapiens OX=9606 GN=IL6R PE=1 SV=1	4.40	1.34

Supplementary Material Table ***S*1** - Proteomics Analysis: Access, Peptides, Scores and Normalized Abundances

**A0A075B6S6**	2 (2)	12.90	2.67e-003	2.28	Immunoglobulin kappa variable 2D-30 OS=Homo sapiens OX=9606 GN=IGKV2D-30 PE=3 SV=1	0.49	0.21
**P01611**	2 (0)	12.71	---	---	Immunoglobulin kappa variable 1D-12 OS=Homo sapiens OX=9606 GN=IGKV1D-12 PE=1 SV=2	---	---

Supplementary Material Table S1 - Proteomics Analysis: Access, Peptides, Scores and Normalized Abundances

**P02750**	2 (2)	12.48	0.09	1.07	Leucine-rich alpha-2-glycoprotein OS=Homo sapiens OX=9606 GN=LRG1 PE=1 SV=2	1.37	1.28
**Q96D15**	2 (2)	12.41	0.43	1.04	Reticulocalbin-3 OS=Homo sapiens OX=9606 GN=RCN3 PE=1 SV=1	1.63	1.70

Supplementary Material Table S1 - Proteomics Analysis: Access, Peptides, Scores and Normalized Abundances

**O14990**	2 (1)	12.39	0.85	1.24	Protein phosphatase inhibitor 2 family member C OS=Homo sapiens OX=9606 GN=PPP1R2C PE=1 SV=1	0.04	0.04
**A0A0C4DH72**	2 (1)	12.00	0.94	1.09	Immunoglobulin kappa variable 1-6 OS=Homo sapiens OX=9606 GN=IGKV1-6 PE=3 SV=1	4.05	3.71

Supplementary Material Table S1 - Proteomics Analysis: Access, Peptides, Scores and Normalized Abundances

**Q92599**	2 (2)	11.48	0.05	1.23	Septin-8 OS=Homo sapiens OX=9606 GN=SEPTIN8 PE=1 SV=4	1.14	1.41
**Q9BR84**	2 (2)	11.29	8.05e-003	1.33	Zinc finger protein 559 OS=Homo sapiens OX=9606 GN=ZNF559 PE=1 SV=1	2.98	2.25
**P24071**	2 (2)	11.01	0.02	1.37		2.86	2.08

Supplementary Material Table *S*1 - Proteomics Analysis: Access, Peptides, Scores and Normalized Abundances

					Immunoglobulin alpha Fc receptor OS=Homo sapiens OX=9606 GN=FCAR PE=1 SV=1		
**Q5SY68**	2 (2)	9.56	0.07	1.18	Protein S100-A7-like 2 OS=Homo sapiens OX=9606 GN=S100A7L2 PE=1 SV=1	1.34	1.14

Supplementary Material Table S1 - Proteomics Analysis: Access, Peptides, Scores and Normalized Abundances

**A0A075B6H7**	1 (1)	7.99	0.01	1.37	Probable non-functional immunoglobulin kappa variable 3-7 OS=Homo sapiens OX=9606 GN=IGKV3-7 PE=1 SV=1	11.30	8.24
**A0A075B6I9**	1 (1)	7.62	0.09	1.30	Immunoglobulin lambda variable 7-46 OS=Homo sapiens OX=9606 GN=IGLV7-46 PE=3 SV=4	8.74	11.34

Supplementary Material Table S1 - Proteomics Analysis: Access, Peptides, Scores and Normalized Abundances

**Q9H977**	1 (1)	7.42	2.59e-003	2.82	WD repeat-containing protein 54 OS=Homo sapiens OX=9606 GN=WDR54 PE=1 SV=1	1.41	0.50
**Q5EBL8**	1 (1)	6.96	0.74	1.11	PDZ domain-containing protein 11 OS=Homo sapiens OX=9606 GN=PDZD11 PE=1 SV=2	0.70	0.78

Supplementary Material Table S1 - Proteomics Analysis: Access, Peptides, Scores and Normalized Abundances

**P02747**	1 (1)	6.92	1.75e-003	1.35	Complement C1q subcomponent subunit C OS=Homo sapiens OX=9606 GN=C1QC PE=1 SV=3	2.20	1.63
**P62820**	1 (0)	6.86	---	---	Ras-related protein Rab-1A OS=Homo sapiens OX=9606 GN=RAB1A PE=1 SV=3	---	---

Supplementary Material Table ***S*****1** - Proteomics Analysis: Access, Peptides, Scores and Normalized Abundances

**Q96IW2**	1 (0)	6.84	---	---	SH2 domain-containing adapter protein D OS=Homo sapiens OX=9606 GN=SHD PE=1 SV=1	---	---
**Q9BV35**	1 (1)	6.75	5.22e-003	2.62	Calcium-binding mitochondrial carrier protein SCaMC-3 OS=Homo sapiens OX=9606 GN=SLC25A23 PE=1 SV=2	0.10	0.04

**Q9Y5W9**	1 (1)	6.68	0.04	2.87	Sorting nexin-11 OS=Homo sapiens OX=9606 GN=SNX11 PE=1 SV=2	2.07	0.72
**P11831**	1 (1)	6.66	8.42e-004	1.27	Serum response factor OS=Homo sapiens OX=9606 GN=SRF PE=1 SV=1	4.12	3.24

Supplementary Material Table S1 - Proteomics Analysis: Access, Peptides, Scores and Normalized Abundances

**Q6UXB3**	1 (0)	6.53	---	---	Ly6/PLAUR domain-containing protein 2 OS=Homo sapiens OX=9606 GN=LYPD2 PE=1 SV=1	---	---
**Q15771**	1 (1)	6.46	0.11	2.02	Ras-related protein Rab-30 OS=Homo sapiens OX=9606 GN=RAB30 PE=1 SV=2	1.57	3.17

Supplementary Material Table S1 - Proteomics Analysis: Access, Peptides, Scores and Normalized Abundances

**Q96SZ5**	1 (1)	6.34	0.55	1.22	2-aminoethanethiol dioxygenase OS=Homo sapiens OX=9606 GN=ADO PE=1 SV=2	20.62	16.87
**Q3LI54**	1 (1)	6.28	9.63e-003	3.21	Keratin-associated protein 19-8 OS=Homo sapiens OX=9606 GN=KRTAP19-8 PE=3 SV=1	0.67	0.21

Supplementary Material Table S1 - Proteomics Analysis: Access, Peptides, Scores and Normalized Abundances

**O60248**	1 (0)	6.26	---	---	Protein SOX-15 OS=Homo sapiens OX=9606 GN=SOX15 PE=1 SV=1	---	---
**P30047**	1 (1)	6.25	6.81e-003	1.65	GTP cyclohydrolase 1 feedback regulatory protein OS=Homo sapiens OX=9606 GN=GCHFR PE=1 SV=3	10.34	6.26

Supplementary Material Table *S*1 - Proteomics Analysis: Access, Peptides, Scores and Normalized Abundances

**P53602**	1 (0)	6.25	---	---	Diphosphomevalonate decarboxylase OS=Homo sapiens OX=9606 GN=MVD PE=1 SV=1	---	---
**P98077**	1 (0)	5.99	---	---	SHC-transforming protein 2 OS=Homo sapiens OX=9606 GN=SHC2 PE=1 SV=4	---	---

Supplementary Material Table S1 - Proteomics Analysis: Access, Peptides, Scores and Normalized Abundances

**Q8IVL5**	1 (1)	5.62	0.74	1.11	Prolyl 3-hydroxylase 2 OS=Homo sapiens OX=9606 GN=P3H2 PE=1 SV=1	12.87	11.61
**O94810**	1 (0)	5.62	---	---	Regulator of G-protein signaling 11 OS=Homo sapiens OX=9606 GN=RGS11 PE=1 SV=2	---	---

Supplementary Material Table S1 - Proteomics Analysis: Access, Peptides, Scores and Normalized Abundances

**Q03591**	1 (1)	5.55	0.04	1.37	Complement factor H-related protein 1 OS=Homo sapiens OX=9606 GN=CFHR1 PE=1 SV=2	0.56	0.41
**A6NDY0**	1 (1)	5.53	0.42	1.31	Embryonic polyadenylate-binding protein 2 OS=Homo sapiens OX=9606 GN=PABPN1L PE=2 SV=1	1.26	1.65

Supplementary Material Table S1 - Proteomics Analysis: Access, Peptides, Scores and Normalized Abundances

**P18615**	1 (1)	5.32	0.25	1.47	Negative elongation factor E OS=Homo sapiens OX=9606 GN=NELFE PE=1 SV=3	0.21	0.30
